# Impacts of the psychological stress response on nonsuicidal self-injury behavior in students during the COVID-19 epidemic in China: the mediating role of sleep disorders

**DOI:** 10.1186/s40359-022-00789-6

**Published:** 2022-04-04

**Authors:** Jiayi Xiao, Ruotong Wang, Yan Hu, Tingxin He, Zhongqiang Ruan, Qi Chen, Ziwen Peng

**Affiliations:** Center for the Study of Applied Psychology, Guangdong Key Laboratory of Mental Health and Cognitive Science and School of Psychology, South China Normal University, Guangzhou, China

**Keywords:** Psychological stress response, Sleep disorders, Emotional management ability, Nonsuicidal self-injury behavior, COVID-19

## Abstract

**Background:**

The sudden outbreak of COVID-19 had a great impact on the physical and mental health of people all over the world, especially for students whose physical and mental development was not yet mature. In order to understand the physical and mental conditions of students during the epidemic period and provide a theoretical basis for coping with psychological problems in public health emergencies, this study explored the mediating role of sleep disorders in the effect of the psychological stress response (PSR) on non-suicidal self-injury (NSSI), along with the moderating role of emotional management ability (EMA).

**Methods:**

The SRQ-20, Pittsburgh Sleep Quality Index, NSSI Behavior Questionnaire, and Emotional Management Questionnaire were used to investigate the mental health of Chinese students in April 10–20 (Time point 1, T1) and May 20–30 (Time point 2, T2), 2020. A total of 1,955 students (Mage = 19.64 years, 51.4% male) were examined at T1 and 342 students (Mage = 20.06 years, 48.2% male) were reassessed at T2.

**Results:**

Overall, the detection rate of PSR and NSSI were 17.60% (n = 344) and 24.90% (n = 486) respectively in the T1 sample, and were 16.37% (n = 56) and 25.44% (n = 87), in the T2 sample. We also found that sleep disorders played a mediating role in the effect of PSR on NSSI in the T1 and T2 samples. In addition, EMA was shown to regulate the effect of PSR on sleep disorders and the effect of sleep disorders on NSSI in the T1 samples.

**Conclusion:**

We found that PSR resulting from public health emergency might lead to NSSI behaviors in individuals. PSR may also cause sleep disorders, which can bring about NSSI. However, these effects were also moderated by the EMA. This research expands our understanding of PSR and NSSI in students during the pandemic.

## Background

The emergence of coronavirus disease 2019 (COVID-19) in December 2019 was followed by a worldwide spread of this difficult to control disease [1]. To curb the spread of COVID-19, protective measures such as lockdowns and quarantines were implemented. This highly contagious disease and the accompanying dramatic lifestyle changes have had a great physical and mental influence on the public [2, 3].

Numerous studies found significant increases in the incidence of anxiety and depression during COVID-19 [4–6]. A meta-analysis of 12 cross-sectional studies published between January 1, 2020 and May 8, 2020 found that the prevalence of depression ranged from 7.45% to 48.30%, and the pooled prevalence of depression was 7 times higher (25%) than a global prevalence estimate (3.44%) of depression in 2017 [7]. Studies have also reported higher levels of sleep disturbances and suicidal thoughts [8, 9]. A study compared the differences in stress responses between students and employees during the epidemic and found that students had overall higher levels of stress response, anxiety and depression than employees [10].

During the epidemic, the stress of quarantine and the academic pressure caused by the suspension of classes made students more vulnerable to physical and mental problems. A survey published by Current Biology Magazine in June 2020 found that students’ sleep start times were generally delayed for every day of the week after the start of online classes [11]. Generally, later sleep timing is associated with poor health outcomes [12]. Furthermore, isolation resulted in a reduction in the amount of physical exercise and an increase in the time spent on electronic devices among students [13]. It was found that young people who followed updates on COVID-19 for more than three hours a day, either through the news or by mobile devices, had higher levels of anxiety and depression, causing changes in a normal biological sleep rhythm [14]. At the same time, students lack experience and have less ability to deal with crises, resulting in students being prone to nonsuicidal self-injury (NSSI), suicide and other problems. Previous studies have shown that NSSI increases the likelihood of suicidal ideation and is a stronger predictor of suicide attempts than other risk factors for suicide (e.g., depression) [15–17]. Our research focused on NSSI during the epidemic and the factors that affected it.

NSSI is most commonly described as deliberate, direct destruction or alteration of body tissue without conscious suicidal intent [18]. Notably, although NSSI refers to behaviors explicitly without suicidal intention, NSSI is associated with suicidal thoughts and behaviors. People who engage in NSSI are approximately four times more likely to subsequently attempt suicide [19]. NSSI is often accompanied by symptoms of depression and anxiety [20, 21]. Some researchers believe that NSSI is a way of coping with negative emotions. Individuals in a state of severe depression can induce positive experiences through NSSI, or they can eliminate negative emotions/cognitive states through NSSI [22]. It is estimated that approximately 4% of the population has a history of NSSI, with higher rates ranging from 14 to 56% in adolescents and 14% to 38% in college students [23, 24].

The psychological stress response (PSR) refers to the processes of adaptive or coping responses made by individuals when they face danger and is a comprehensive set of responses that occurs when individuals deal with changes in the internal and external environment [25]. Previous studies found that in previous epidemics of infectious diseases such as the severe acute respiratory syndrome (SARS) epidemic, people suffered from the psychological stress response [26, 27].Significant environmental changes during the epidemic have led to increased levels of psychological stress [28, 29]. When an individual cannot effectively cope with the stress caused by an emergency, self-injury is more likely to occur [30, 31]. Measurements of skin conductivity and cortisol have shown that self-injurers had an increased response to stressful events, exhibited higher physiological arousal, and had less cortisol secretion [32, 33].

Sleep disorders are also an important predictor of suicidal behavior and ideation [34, 35]. Sleep disturbances can increase the risk of suicide by 1.95 to 2.95 times [36]. The PSR has been associated with sleep inhibition and increased arousal, which may lead to sleep disorders [37]. Individuals experiencing high levels of stress are more likely to suffer from sleep disorders [38]. Adolescents with high levels of a PSR and sleep deprivation are more likely to have suicidal behavior or ideation [39]. Therefore, we speculate that a high PSR can increase the risk of sleep disorders, suicide and NSSI. Sleep disorders may also increase the risk of suicide or NSSI. The influence of PSR on NSSI may be partially mediated by sleep disorders [22]. However, few studies have explored and verified this relationship.

Emotional management ability (EMA) is the ability to correctly recognize one's own and others' emotions and purposefully guide, regulate, and control one's own emotions, ultimately allowing for healthy emotional development [40]. According to the Experiential Avoidance Model (EAM), individuals who lack strategies of emotion control or methods of emotion regulation may adopt NSSI as a means of emotion regulation [41]. Studies have found that an important function of NSSI is to release, express and transmit emotional feelings, and therefore, emotional management disorders are related to NSSI. Meanwhile, EMA can also have an effect on one's sleep. If individuals who are subjected to stressful events cannot adopt positive and rational coping strategies to regulate their emotions, it will lead to continuous arousal and dysregulation of the physiological components in the emotional response system, thus producing sleep problems [42–44].

In this study, we investigated a moderated mediation model among a large sample of Chinese adolescents and college students to understand the relationships among PSR, sleep disorders, EMA, and NSSI. Based on previous research, we propose three hypotheses: (1) PSR is positively related to NSSI. (2) Sleep disorders will mediate the association between PSR and NSSI. (3) EMA will moderate the mediation model; that is, EMA will regulate the effects of PSR on sleep disorders and the effects of sleep disorders on NSSI behavior.

## Methods

### Participants and procedure

Two online tests were conducted to assess the mental health status of students from April 10–20 (time point 1; T1) and May 20–30 (time point 2; T2), 2020. We invited students in China to participate in the investigation through the Wenjuanxing online survey platform (https://www.wjx.cn/). Only one response can come from each IP address. The network automatically records the response time to complete the questionnaire. To ensure the feasibility and validity of the questionnaire, a response time of less than 100 s was regarded as invalid. After eliminating the invalid questionnaires, 1,955 participants were included at T1. Of these, 420 participants were willing to be involved in the second investigation and provided contact information. Valid questionnaires were collected from 342 of these participants at T2. The protocol was approved by the Institutional Research and Ethics Committee of South China Normal University (SCNU-PSY-2020–1-059) in accordance with the 1964 Helsinki Declaration and its later amendments or comparable ethical standards. Informed consent was obtained from all subjects and/or their legal guardian.

### Self-report measures

The Chinese version of the 20-item self-report questionnaire (SRQ-20) was used to assess the PSR. This measure was incorporated into the *Disaster Psychological Crisis Intervention Training Manual* and has relatively high validity in measuring mental health problems [45]. The SRQ-20 is used to measure an individual's psychological response, including depression or anxiety, to crisis events. The questions were rated on a two-point scale including 0 = no and 1 = yes. Higher total scores are indicative of a higher psychological stress response. A total score greater than 8 indicates a significant PSR. The Cronbach’s α value of the SRQ-20 in the present study was 0.92 (T1 and T2).

NSSI was assessed by the Chinese version of an 8-item self-report questionnaire. The respondents were asked “Have you harmed yourself in some way in the past year? Yes/No”. A list of eight behaviors (hitting, pulling hair, banging head, pinching, scratching, biting, exposure to flame/burning, cutting) was then presented. The frequency of various NSSI behaviors was summed, and a higher frequency indicated more serious NSSI. If the overall frequency of NSSI is more than 1, it is believed that NSSI exists [46]. In the current study, this questionnaire had Cronbach's α values of 0.80 (T1) and 0.68 (T2).

We used the sleep disorders subscale from the Pittsburgh Sleep Quality Index to measure the sleep disorders of the students. The scale contains 9 items and adopts a four-point score (1 = no to 4 = more than 3 times a week). A higher total score of all items indicated that individuals had more severe sleep disturbances. The Cronbach’s α values of this scale in the present study were 0.95 (T1) and 0.85 (T2).

The subscale of emotion management factors in the emotional intelligence scale compiled by Goleman (1995) was used to measure whether individuals can effectively and appropriately detect, control, vent and regulate their negative emotions. The scale contains 4 items and adopts a four-point score (1 = always to 4 = never). A higher total score of all items indicated that individuals had a stronger ability to manage their emotions. The Cronbach’s α value of this scale in the present study was 0.85 (T1 and T2).

### Data analysis

We first conducted descriptive analyses by examining the bivariate correlations between all study variables using SPSS 23. Second, the analyses of moderated mediation at T1 were constructed using Hayes’s (2013) PROCESS macro (Model 58, a mediated mediation model). Finally, we replicated the moderated mediation analysis at T2.

A bootstrapping procedure with 5000 iterations was used to examine the significance of all the effects to obtain robust standard errors for parameter estimation, which is a common method for significance testing in mediation analysis. A 95% bias-corrected (BC) confidence interval (95% CI) for each of these effects was produced. CIs that did not contain zero were an indication that the effects were significant. All continuous variables were standardized (Z-scores), and the interaction terms were computed from these standardized scores.

## Results

### Common method bias test

In this study, anonymous measurement and reverse scoring were used to control for common method deviation. The Harman single-factor test method was used to test the common method deviation of the collected data. Additionally, we performed factor analysis of all items on the four questionnaires (T1: KMO = 0.96, Bartlett = 38,822.944, *df* = 780, *p* < 0.001; T2: KMO = 0.92, Bartlett = 6051.246, *df* = 780, *p* < 0.001). A total of 6 (T1) and 8 (T2) factors with characteristic roots greater than 1 were extracted, and the maximum factor variance interpretation rates were 30.70% (T1) and 30.92% (T2) (both less than 40%). Therefore, there was no serious common method deviation in this study.

### Descriptive analyses

#### Demographic variables

The sociodemographic characteristics of the sample at T1 and T2 are shown in Table 1. The mean age of the participants in the T1 sample was 19.64 years (*SD* = 4.04). The gender distribution was roughly equal, with 51.40% males and 48.60% females. There were 51.90% adolescents and 48.10% undergraduates, with 17.70% being from the high-risk COVID-19 area (Hubei). In this sample, 80.40% of the students studied at home.

**Table 1:** Sociodemographic characteristics of the sample

	T1	T2
	N = 1955 (%)	N = 342 (%)
Grade		
Adolescent	1015 (51.90)	84 (24.56)
Undergraduate	940 (48.10)	258 (75.44)
Gender		
Male	1005 (51.40)	165 (48.20)
Female	950 (48.60)	177 (51.80)
Area		
High-risk area of COVID-19	347 (17.70)	57 (16.67)
Low-risk area of COVID-19	1608 (82.30)	285 (83.33)
Current state		
Quarantine in a medical facility	26 (1.30)	5 (1.48)
Compulsory quarantine at home or in a specific location	148 (0.72)	27 (7.89)
Learning at home	1572 (80.40)	166 (48.54)
Resume normal learning	209 (10.69)	144 (42.11)
Only-child family		
Yes	1106 (55.90)	198 (57.89)
No	870 (44)	144 (42.11)
Registered residence		
City	1164 (59.50)	187 (54.68)
Country	791 (40.50)	155 (45.32)

In the T2 sample, the mean age of the participants was 20.06 years (*SD* = 3.98). There were 48.20% males and 51.80% females, 24.56% adolescents and 75.44% undergraduates, and 16.67% being from the high-risk COVID-19 area (Hubei). Students continuing to learn at home and those who had resumed learning at school accounted for 48.54% and 42.11%, respectively.

#### Detected rates of PSR and NSSI

In the T1 sample, the detected rates of PSR and NSSI were 17.60% (*n* = 344) and 24.90% (*n* = 486), respectively. The students in high-risk areas reported significantly higher PSR (32.28%) and NSSI (37.75%) than those in low-risk areas (PSR: 14.43% and NSSI: 22.08%) (PSR: χ^2^ = 62.71, *df* = 1, *p* < 0.001; NSSI: χ^2^ = 37.54, *df* = 1, *p* < 0.001). The detected rates of PSR and NSSI in the T2 sample were 16.37% (*n* = 56) and 25.44% (*n* = 87), respectively. The students in high-risk areas (31.58%) reported a significantly higher PSR than those in low-risk areas (16.14%) (χ^2^ = 7.44, *df* = 1, *p* < 0.01). Paired sample T test showed no significant difference in PSR (*t*_T1-T2_ =  − 0.2, *p* = 0.842), Sleep disorders (*t*_T1-T2_ = 0.461, *p* = 0.645) and NSSI (*t*_T1-T2_ = -0.089, *p* = 0.929) at T1 and T2, but EMA decreased significantly (*t*_T1-T2_ = 2.675, *p* = 0.008) in T2. ANOVA results showed that there were differences between groups in gender, age, area, current student status and monthly household income (*p* < 0.05) in NSSI, so we controlled for these variables in the subsequent model.

#### Partial correlation analyses

Table 2 presents descriptive statistics for all variables, including the mean, standard deviation, skewness, and kurtosis, as well as partial correlations between these variables after controlling for variables such as gender, age, area, current status and monthly household income. PSR was positively correlated with sleep disorders and NSSI but negatively correlated with EMA in the T1 sample. EMA was negatively correlated with sleep disorders and NSSI. Sleep disorders were positively correlated with NSSI in the T1 sample. However, in the T2 sample, NSSI was not significantly associated with PSR or EMA. In the subsequent analyses, we focused on the meditating effects of sleep disorders. Considering that the correlation coefficient between PSR and EMA is greater than 0.6 at two time points, we used variance inflation factor (VIF) to test whether multicollinearity exists. The regression equation was established with PSR, EM, NSSI or sleep disorder as dependent variables and residual variables as independent variables, the VIF were all less than 2, indicating the absence of multicollinearity.

**Table 2:** Partial correlation analyses and descriptive statistics of the study variables

	T1 (*N* = 1955)				T2 (*N* = 342)			
Variable	1	2	3	4	1	2	3	4
1. PSR	1				1			
2. EMA	− 0.63***	1			− 0.66***	1		
3. Sleep disorders	0.43***	− 0.38***	1		0.46***	− 0.42***	1	
4. NSSI	0.53***	− 0.39***	0.27***	1	0.07	− 0.04	0.12*	1
*M*	3.36	11.85	1.01	0.58	3.99	11.30	1.06	0.26
*SD*	4.59	2.86	0.78	1.27	4.90	2.91	0.69	0.45
Skewness	1.53	− 0.47	0.57	2.79	1.38	− 0.22	0.41	1.29
Kurtosis	1.82	− 0.16	0.10	8.35	1.22	− 0.55	0.39	0.17

*PSR* psychological stress response; *EMA* emotional management ability; *NSSI* nonsuicidal self-injury

^*^p < 0.05, **p < 0.01, ***p < 0.001

### Mediating effect of sleep disorders in the T1 sample

We used Model 4 in the PROCESS macro to test the mediating effect of sleep disorders on the relationship between PSR and NSSI. The results (Table 3) showed that PSR was positively associated with sleep disorders (β = 0.68, *p* < 0.001, 95% CI: 0.59, 0.76). Sleep disorders were positively related to NSSI (β = 0.19, *p* < 0.001, 95% CI: 0.13, 0.24). PSR had indirect effects on NSSI through sleep disorders (β = 0.99, *p* < 0.001, 95% CI: 0.88, 1.10). There were two paths by which the PSR affected NSSI (Table 4). First, the PSR affected NSSI directly, and this direct effect accounted for 88.60% of the total effect. Second, the PSR affected NSSI indirectly via sleep disorders, and this indirect effect accounted for 11.40% of the total effect.

**Table 3:** The mediation effect size of sleep disorders in the relationship between PSR and NSSI in the T1 sample

Variable	Model 1 (NSSI)		Model 2 (sleep disorders)		Model 3 (NSSI)	
	β	*t*	β	*t*	β	*t*
PSR	1.12	20.79***	0.68	15.56***	0.99	17.57***
Sleep disorders					0.19	6.80***
Gender	− 0.05	− 1.11	− 0.10	− 3.10**	− 0.03	− 0.65
Age	0.01	2.67**	0.01	3.08**	0.01	2.22*
Monthly household income	− 0.00	− 0.22	0.01	0.56	− 0.01	− 0.31
Area	− 0.20	− 3.68***	− 0.15	− 3.40***	− 0.17	− 3.18**
Current status	− 0.08	− 1.90	− 0.17	− 4.76***	− 0.05	− 1.18
*R*	0.46		0.40		0.48	
*R*^2^	0.22		0.16		0.23	
*F* (*df*)	89.08*** (6, 1948)		63.17*** (6, 1948)		84.74*** (7, 1947)	

*PSR* psychological stress response; *EMA* emotional management ability; *NSSI* nonsuicidal self-injury

^*^p < 0.05, **p < 0.01, ***p < 0.001

**Table 4:** The mediation effect size of sleep disorders in the relationship between PSR and NSSI in the T1 sample

Path	Effect	Boot SE	Boot 95% CI		P_m_%
			LL CL	UL CL	
PSR → NSSI	0.99	0.06	0.88	1.10	88.60%
PSR → Sleep Disorders → NSSI	0.13	0.03	0.08	0.18	11.40%
Total	1.12	0.05	1.01	1.22	

*PSR* psychological stress response; *NSSI* nonsuicidal self-injury

### Moderated mediation model in the T1 sample

**Fig. 1:**
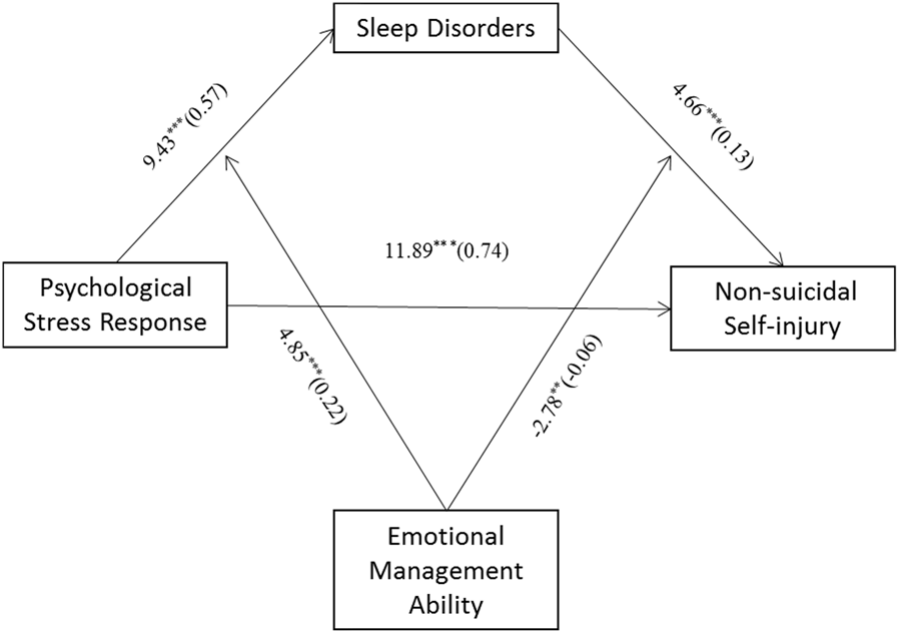
The moderated mediation model in the T1 sample. Sleep disorder plays a mediating role in the relationship between PSR and NSSI, and EMA plays a mediating role in the relationship between PSR, sleep disorders and NSSI in the T1 sample. *p < 0.05, **p < 0.01, ***p < 0.001

We tested the moderated mediation model with the latent interaction of EMA and sleep disorders (Fig. 1). PSR still had a positive association with sleep disorders (β = 0.57, *p* < 0.001, 95% CI: 0.45, 0.69), while sleep disorders were positively related to NSSI (β = 0.13, *p* < 0.001, 95% CI: 0.08, 0.19). The PSR had a partially indirect effect on NSSI through sleep disorders (β = 0.74, *p* < 0.001, 95% CI: 0.61, 0.86). Moreover, the interaction between PSR and EMA had a significant predictive effect on sleep disorders (β = 0.22, *p* < 0.001, 95% CI: 0.13, 0.31), indicating that the predictive effect of PSR on sleep disorders was regulated by EMA. The interaction between sleep disorders and EMA had a significant predictive effect on NSSI (β = -0.06, *p* < 0.01, 95% CI: − 0.11, − 0.02), indicating that the predictive effect of sleep disorders on NSSI was regulated by EMA (Table 5).

**Table 5:** Testing the moderated mediating effect of PSR on NSSI in the T1 sample

Regression equation (*N* = 1955)		Fitting index			Coefficient significance	
Outcome variable	Predictor variable	*R*	*R*^2^	*F (df)*	*β*	*t*
Sleep disorders		0.48	0.23	72.93 (8, 1946)		
	PSR				0.57	9.43***
	EMA				− 0.26	− 13.05***
	PSR*EMA				0.22	4.85***
	Gender				− 0.11	− 3.55***
	Age				0.02	4.05***
	Area				− 0.14	− 3.36***
	Monthly household income				0.01	0.70
	Current status				− 0.17	− 4.92***
NSSI		0.52	0.27	78.36 (9, 1945)		
	PSR				0.74	11.89***
	Sleep disorders				0.13	4.66***
	EMA				− 0.14	− 4.19***
	Sleep disorders*EMA				− 0.06	− 2.78**
	Gender				− 0.04	− 1.01
	Age				0.02	3.09**
	Area				− 0.18	− 3.34***
	Monthly household income				− 0.01	− 0.40
	Current status				− 0.06	− 1.35

*PSR* psychological stress response; *EMA* emotional management ability; *NSSI* nonsuicidal self-injury

^*^p < 0.05, **p < 0.01, ***p < 0.001

**Fig. 2:**
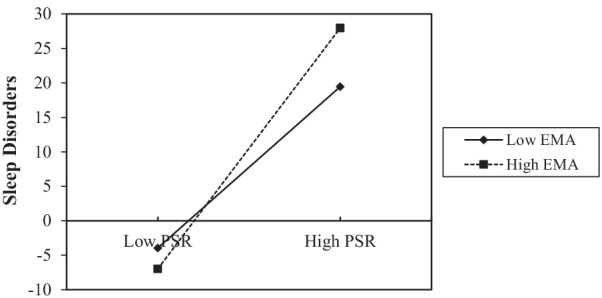
Regulating effect in the T1 sample. The effect of PSR on sleep disorders was regulated by different levels of EMA

**Fig. 3:**
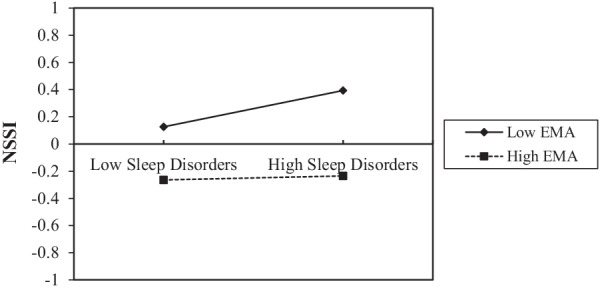
Regulating effect in the T1 sample. The effect of sleep disorders on NSSI was regulated by different levels of EMA

To further describe the effect of moderating variables, a simple slope graph was drawn for low and high levels of EMA (M ± 1 SD). Figure 2 intuitively shows how the effects of the PSR on sleep disorders were regulated by different levels of EMA. PSR had a weaker positive association with sleep disorders when EMA was low (M-1 SD; simple slope = 0.35, *p* < 0.001) than when EMA was high (M + 1 SD; simple slope = 0.81, *p* < 0.001). Figure 3 shows how the effects of sleep disorders on NSSI were regulated by different levels of EMA. Among the students with low EMA (M-1 SD), sleep disorders had a positive predictive effect on NSSI (M-1 SD; simple slope = 0.19, *p* < 0.001). For students with high EMA (M + 1 SD), sleep disorders had no significant positive predictive effect on NSSI (M-1 SD; simple slope = 0.06, *p* = 0.0783). The conditional indirect effect of PSR on NSSI through sleep disorders modulated by different EMA levels is tabulated in Table 6. A weaker indirect effect of sleep disorders was seen with high EMA (β = 0.069, 95% CI = 0.017, 0.132) than with low EMA (β = 0.051, 95% CI = -0.006, 0.120). These results verified the moderating effects of EMA on the relationship between PSR and NSSI via sleep disorders.

**Table 6:** The conditional indirect effect of PSR on NSSI through sleep disorders for different EMA levels in the T1 sample

Conditional effect of EMA	Effect	Boot SE	Boot 95% CI
Low (*M-1 SD*)	0.069	0.029	(0.017, 0.132)
Medium (*M*)	0.075	0.022	(0.034, 0.120)
High (*M* + *1 SD*)	0.051	0.031	(− 0.006, 0.120)

*PSR* psychological stress response; *EMA* emotional management ability; *NSSI* nonsuicidal self-injury

### Mediating effect of sleep disorders in the T2 sample

**Fig. 4:**
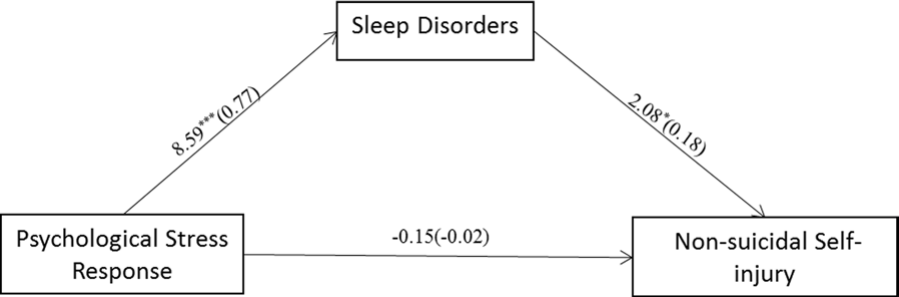
The mediating effect of sleep disorders in the T2 sample. Sleep disorders played a mediating role in the relationship between PSR and NSSI in the T2 sample. *p < 0.05, **p < 0.01, ***p < 0.001

We also used Model 4 in the PROCESS macro to test the mediating effect of sleep disorders on the relationship between PSR and NSSI in the T2 sample (Fig. 4). The results (Table 7) again showed that PSR had a positive effect on sleep disorders (β = 0.77, *p* < 0.001, 95% CI: 0.59, 0.94). Sleep disorders were positively related to NSSI (β = 0.18, *p* < 0.05, 95% CI: 0.01, 0.36). PSR had no significant direct effects on NSSI (β = -0.02, *p* = -0.15, 95% CI: − 0.34, 0.29). Therefore, the direct effect of PSR on NSSI accounted for 21.17% of the total effect, while the indirect effect accounted for 78.83% of the total effect (Table 8). However, we did not find a moderating effect of EMA.

**Table 7:** The mediation effect size of sleep disorders in the relationship between PSR and NSSI in the T2 sample

Variable	Model 1 (NSSI)		Model 2 (sleep disorders)		Model 3 (NSSI)	
	β	*t*	β	*t*	β	*t*
PSR	0.12	0.80	0.77	8.59***	− 0.02	− 0.15
Sleep disorders					0.18	2.08*
Gender	− 0.04	− 0.39	− 0.12	− 1.66	− 0.02	− 0.21
Age	− 0.02	− 1.05	0.01	1.07	− 0.02	− 1.17
Monthly household income	0.03	0.55	0.00	0.08	0.03	0.54
Area	0.03	0.19	0.15	0.90	0.01	0.09
Current status	0.09	0.94	− 0.03	− 0.48	0.10	1.00
*R*	0.10		0.47		0.15	
*R*^2^	0.01		0.22		0.02	
*F* (*df*)	0.59 (6, 325)		15.72*** (6, 335)		1.13 (7, 334)	

*PSR* psychological stress response; *EMA* emotional management ability; *NSSI* nonsuicidal self-injury

^*^p < 0.05, **p < 0.01, ***p < 0.001

**Table 8:** The mediation effect size of sleep disorders in the relationship between PSR and NSSI in the T2 sample

Path	Effect	Boot SE	Boot 95% CI		P_m_%
			LL CL	UL CL	
PSR → NSSI	− 0.02	0.16	− 0.34	0.29	21.17%
PSR → Sleep disorders → NSSI	0.14	0.07	0.01	0.29	78.83%
Total	0.12	0.15	− 0.17	0.40	

*PSR* psychological stress response; *NSSI* nonsuicidal self-injury

## Discussion

In this study, the psychological and physiological states of students during the Covid-19 epidemic were investigated through data collection at two time points. Based on previous studies, the relationships between PSR, sleep disorders, EMA and NSSI were examined, and mediating and moderating mediating models were constructed.

We found that the detected rates of PSR and NSSI were 17.60% and 24.90%, respectively, in the T1 sample and 16.37% and 25.44% in the T2 sample. There was no significant difference in the detected rates between the two measurements. However, EMA decreased significantly in T2. This is probably because these two surveys were taken in April and May. The epidemic was still relatively serious in April, leading to PSR and NSSI. Although the epidemic was considered to be under control in May, students were preparing to return to school, which brought new pressure. Therefore, some physical and psychological changes would be relatively stable between these two time points. As for the EMA, according to the three stages of PSR, which include alertness, resistance and exhaustion, after the first four months of alertness and resistance to the epidemic, people may have entered the exhaustion stage in May, consequently EMA declined significantly.

This study also found that the PSR was significantly positively correlated with NSSI, which was consistent with previous research [31, 47]. According to emotional cognitive models, NSSI is an inappropriate method of emotion regulation. When individuals are subjected to acute stress, a series of psychological and physiological disruptions will occur. If individuals are unable to adjust in an appropriate way, NSSI may be adopted [48, 49]. During the epidemic, disease and lockdown triggered anxiety and panic within the population. At the same time, students were forced to change the way they learned. This large number of changes in life and study style resulted in students with a higher PSR. Because of the immature physical and mental development of students, they adopted NSSI as one of the ways to cope with and adjust to the psychological pressure.

Our study found that sleep disorders were positively correlated with the PSR and NSSI. In addition, we found that sleep disorders played a mediating role in the effects of PSR on NSSI. The PSR during the epidemic not only directly affected NSSI but also affected NSSI by causing or worsening sleep disorders. According to previous studies, the hypothalamic–pituitary–adrenal (HPA) axis can regulate the release of glucocorticoids (GCs; mainly cortisol in humans) and the stress response. The dynamic response of the HPA axis to stressors is helpful for individuals to adapt and cope with stress, but long-term excessive activation will have negative effects on the body and mind [50]. Dysfunction of the HPA axis increases the risk of suicide approximately 4.5 times [51]. Overactivation of the HPA axis increases shallow sleep and night wakefulness of individuals, while insufficient sleep increases the basic activities of the HPA axis, inducing neuroendocrine changes and affecting the function of the HPA axis [52, 53]. During the epidemic, the long term PSR overactivated the HPA axis and led to its dysfunction, ultimately causing sleep disorders and NSSI.

We also found that EMA played a regulatory role in the T1 samples. EMA not only regulated the influence of the PSR on sleep disorders but also regulated the influence of sleep disorders on NSSI. Regarding EMA regulation of the effects of the PSR on sleep disorders, the results were somewhat unexpected. This study found that in the face of high PSR, the students with high EMA had more serious sleep disorders than those with low EMA. This is probably because individuals with high EMA were extremely sensitive to stress related to the epidemic and their own emotional state and used more cognitive resources to maintain their physical and mental health. However, they unconsciously fell into another kind of anxiety, causing more serious sleep disorders. Of course, this result has yet to be independently verified. The potential mechanism of this impact also needs to be further investigated. EMA also regulated the impact of sleep disorders on NSSI. For students with low EMA, the more severe the sleep disorders were, the more NSSI was predicted. However, for those with high EMA, sleep disorders no longer had a positive predictive effect on NSSI. Individuals with EMA have more appropriate and flexible ways to vent and regulate emotions, which can manage negative emotions such as anxiety and depression in a timely manner and help reduce the occurrence of NSSI [48]. Students who were not good at regulating their emotions failed to reasonably cope with their emotions. When negative emotions accumulated to a certain level, they were more likely to engage in NSSI and other behaviors as catharsis. Unfortunately, we did not find a regulating effect of EMA in the second measurement. This may be due to the significantly decline in EMA among the students in T2, indicating that EMA were not available to continue to play a regulatory role on sleep and NSSI.

In conclusion, this study found that sleep disorders played a mediating role in the effects of the PSR on NSSI through data collection at two time points. We also tested the moderating role of EMA. However, our study has some shortcomings. First of all, the size of the sample decreased significantly at T2. This was because the data was collected online and only 420 subjects left their contact information and subsequently participated in the second collection. This may affect the reliability of the results of the second data analysis, for example, due to survivor bias. Future research should adopt methods to reduce the attrition rate of subjects. In addition, the self-report questionnaire asked about NSSI in the past year, whereas data collection occurred less than 6 months after the emergence of the COVID-19 disease. Hence, some of the NSSI reported may relate to pre-pandemic period. However, students in high-risk areas reported significantly higher NSSI (37.75%) than those in low-risk areas (22.08%) in T1, which supports the conclusion that the epidemic had a great impact on NSSI. Finally, although we controlled for year of education in our data analysis, participants included high school, middle school and university students, all of whom have different psychological characteristics. Therefore, future research should study these groups separately.

## Conclusion

In this study, it was found that the PSR promoted NSSI during the COVID-19 epidemic, while sleep disorders played a mediating role. The PSR affected NSSI directly, and also affected NSSI by causing or worsening sleep disorders. In the T1 sample, we also found a moderating role of EMA in the effect of the PSR on sleep disorders and sleep disorders on NSSI behavior. However, the significantly decline in EMA among the students in T2 indicates that EMA did not continue to play a regulatory role on sleep and NSSI as the epidemic developed.

In the face of stressful events such as the epidemic, people's mental health needs to be paid attention to, especially for younger students. Individuals’ mental states are affected not only by external stimuli but also by internal physiological states and rhythms. We need to develop our own EMA and learn emotional regulation strategies to help us better regulate our emotions and maintain good sleep quality and psychological states.

## Data Availability

The datasets used and/or analyzed during the current study available from the corresponding author on reasonable request.
